# Thermophilic fermentation enhanced by eggshell-derived hydroxyapatite for sustainable hydrogen production

**DOI:** 10.1186/s40643-025-00976-4

**Published:** 2025-11-21

**Authors:** Shishita Zahan Zisha, Hasfalina Che Man, Rozita Omar, Suraya Abdul Rashid, Tan Jian Ping, Shareena Fairuz, Abdul Manaf, Tabassum Mumtaz, Nur Syakina Jamali

**Affiliations:** 1 Department of Chemical and Environmental Engineering, Faculty of Engineering, Universiti Putra Malaysia, Serdang, 43400 Selangor Malaysia; 2 Nanomaterials Processing and Technology Laboratory (NPTL), Institute of Nanoscience and Nanotechnology, Universiti Putra Malaysia, Serdang, 43400 Selangor Malaysia; 3 School of Energy and Chemical Engineering, Xiamen University Malaysia, Jalan Sunsuria, Bandar Sunsuria, Sepang, 43900 Selangor Malaysia; 4 School of Chemical Engineering, College of Engineering, Universiti Teknologi MARA, 40450 Shah Alam, Selangor Malaysia; 5 Institute of Food and Radiation Biology, Bangladesh Atomic Energy Commission, Dhaka, Bangladesh

**Keywords:** Green synthesis, Hydroxyapatite from eggshells, Optimization using response surface methodology, Stoichiometric ratio, Biohydrogen production

## Abstract

This study presents the eco-friendly synthesis of Hydroxyapatite (HAP) using chicken eggshell biowaste as a sustainable calcium source to enhance biohydrogen production. The stoichiometric ratio of Ca/P was determined to be 1.67 by evaluating pH, temperature, and calcination time, revealing that pH 10.5, temperature 950ᵒC, and calcination time of 2 h were optimal. Structural and morphological characterization confirmed the successful formation of crystalline HAP with 78% crystallinity (XRD), accurate Ca/P ratio (EDS), and phosphate group presence (FTIR). The synthesized HAP was applied as a bio-additive in thermophilic dark fermentation using a *Thermoanaerobacterium*-enriched sludge. The optimal HAP dosage (560 mg/L) yielded the highest hydrogen yield of 1.085 mol H_2_/mol sugar and a hydrogen production rate of 27.969 mL H_2_/(L h), representing a 59.73% increase over the control. Volatile fatty acid analysis and microbial profiling confirmed enhanced butyrate metabolism and a dominant presence of *Thermoanaerobacterium thermosaccharolyticum*. This work demonstrates a novel waste-to-value approach by integrating green-synthesized HAP with thermophilic fermentation, contributing to both waste valorization and renewable hydrogen production.

**Graphical abstract:**

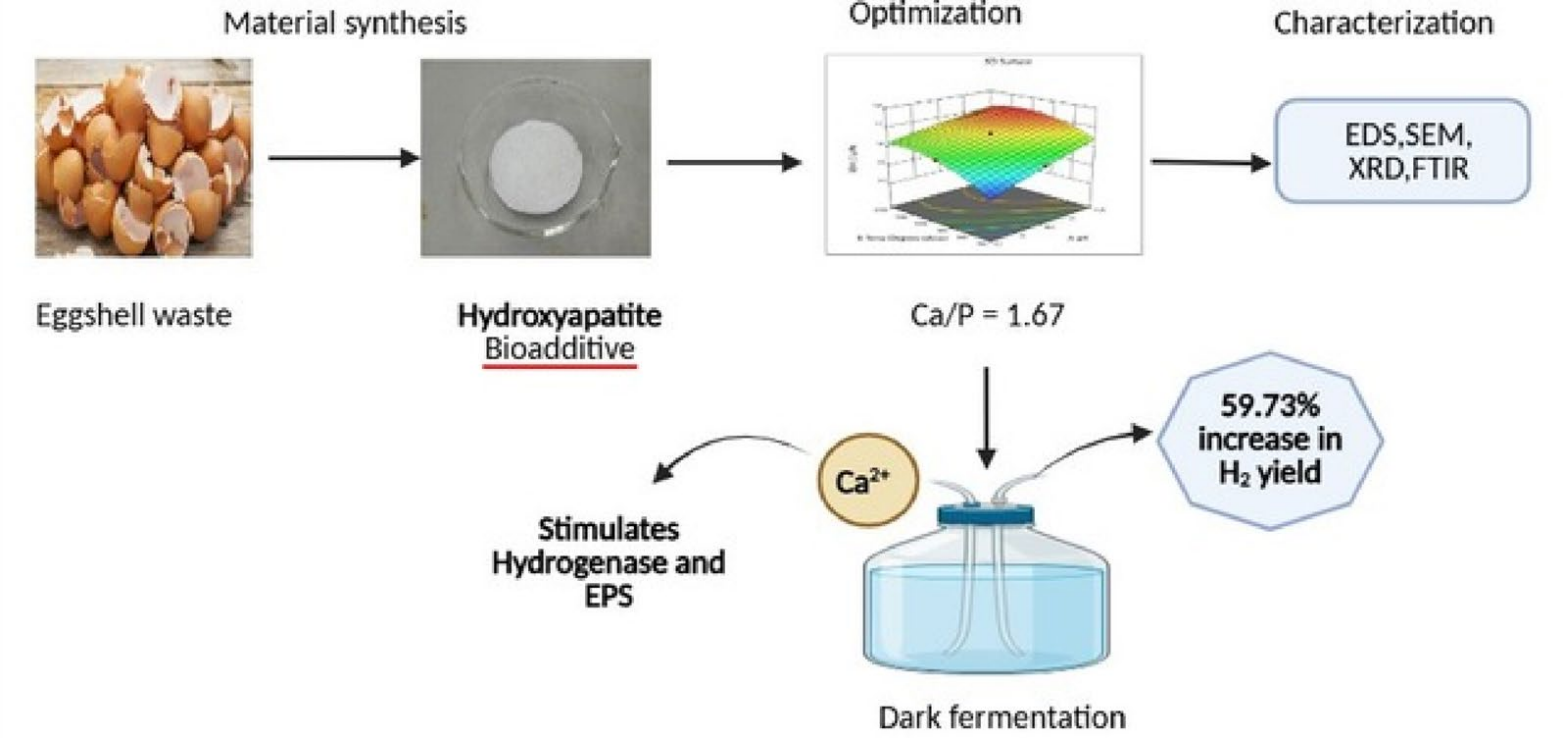

**Supplementary Information:**

The online version contains supplementary material available at 10.1186/s40643-025-00976-4.

## Introduction

The excessive dependence on non-renewable fossil fuels has resulted in critical global challenges, including energy insecurity, environmental degradation, and increased greenhouse gas emissions (Rahman et al. 2020). According to Zhang et al. (2021), the combustion of fossil fuels emits approximately 35 billion tonnes of carbon dioxide (CO_2_) annually, significantly contributing to global warming and acid rain pollution (Younas et al. 2022). These concerns have intensified the search for alternative, sustainable energy sources such as hydrogen, which offers high energy density and emits only water upon combustion. However, approximately 90% of hydrogen is produced using fossil resources, making the process environmentally unsustainable (Wang et al. 2022).

Furthermore, hydrogen can also be produced from renewable resources via biological and physicochemical mechanisms. Among these, biological routes, particularly thermophilic dark fermentation has attracted growing interest due to its moderate operating conditions, simplicity, and ability to convert organic waste into hydrogen (Cheng et al. 2022; Jariyaboon et al. 2023). Recent reviews have highlighted advancements in dark fermentation technology, including microbial enrichment strategies, reactor configurations, and the use of nanomaterials or bio-additives to enhance yield and process stability (Ji and Wang 2021; Wang and Yin 2018; Yang and Wang 2018). These studies emphasize the potential of combining sustainable catalysts with waste-derived materials in hydrogen production systems to enhance efficiency while adhering to green chemistry principles. Thermophilic dark fermentation, in particular, is known for its high microbial activity and accelerated metabolic rates at elevated temperatures. Despite these advantages, this approach also faces considerable limitations, such as the need for specialized high-temperature bioreactors, low hydrogen yields, microbial growth inhibition, and by-product accumulation (Zhang et al. 2019; Jamali et al. 2019a; Fahoul et al. 2022).

To overcome these challenges, researchers have explored the use of bio-additives, particularly catalytic materials that enhance microbial performance. These additives can promote microbial activity and metabolic efficiency by providing a large surface area, acting as electron carriers, or facilitating nutrient exchange (Ramprakash and Incharoensakdi 2020; Yang et al. 2024). Recent attention has turned to green synthesis of such bio-additives to align with sustainability goals using environmentally benign processes and natural waste materials.

In recent years, various bio-additives such as silver, copper, nickel, iron, activated carbon, carbon nanotubes, and composites have been studied for their potential to enhance dark fermentative hydrogen production. Among these, iron and nickel are particularly noteworthy due to their role as essential cofactors in hydrogenase enzymes, thereby enhancing catalytic activity and hydrogen yield (Shanmugam et al. 2020). Light metal ions such as calcium (Ca^2^⁺) have also been associated with enzymatic cofactors and dehydrogenase activity in bacterial systems, further emphasizing their relevance in microbial bioenergy processes (Zhang et al. 2022). The incorporation of calcium-based materials is particularly promising due to their role in modulating microbial performance.

One such promising bio-additive is hydroxyapatite (HAP), a calcium phosphate ceramic (Ca_5_(PO_4_)_3_(OH)) with a stoichiometric Ca/P ratio of 1.67 (Aswathi et al. 2023; Liu et al. 2021). As a biocompatible and bioactive material, HAP has been widely used in biomedical, environmental, and energy sectors (Upadhyay and Ullah 2024). Its structural properties, such as high surface area and bioactivity, promote the production of extracellular polymeric substances (EPS) and facilitate electron transfer in microbial systems, thereby enhancing hydrogen yields in dark fermentation processes (Irwansyah et al. 2022). Importantly, the bioactivity and catalytic function of HAP can be influenced by the presence of trace elements such as magnesium, silicon, strontium, and iron, which are often lacking in conventionally synthesized HAP but can be retained through green synthesis routes (Metco 2018; Hartati et al. 2022; Ma et al. 2020).

Eggshells have emerged as a highly suitable precursor for HAP synthesis among various waste-derived calcium sources due to their high calcium carbonate content. Annually, the global production of chicken eggs could generate between 7.83 and 10.44 million tonnes of eggshell waste (Vigneron and Holanda 2024). Utilizing this abundant waste stream reduces the environmental burden and supports circular resource management by transforming waste into valuable biocatalysts. The resulting HAP is particularly suitable for microbial enhancement in dark fermentation due to its structural and chemical compatibility.

This study employs a waste-to-value strategy to synthesize stoichiometric HAP from locally sourced eggshells, using Response Surface Methodology (RSM) with Central Composite Design (CCD) to optimize pH, calcination temperature, and time. The optimized HAP was applied in thermophilic dark fermentation using *Thermoanaerobacterium*-enriched sludge, and the modified Gompertz model was employed to evaluate hydrogen production kinetics and substrate utilization. This integrated approach advances biohydrogen production, promoting green chemistry and sustainable resource recovery.

## Materials and methods

### Eco-friendly Synthesis of Hydroxyapatite (HAP)

#### Pretreatment

The chicken eggshells were collected from a local food outlet in Kajang. Initially, the shells were washed and then boiled in distilled water at 100 °C for 25–30 min to remove surface impurities. Subsequently, the shells were treated with 1 M acetic acid for 17 min to facilitate inner membrane separation, as described by Aina et al. (2022). After leaching, the shells were thoroughly rinsed with deionized water and dried in an oven at 60 °C overnight.

A total of 20 g of pretreated eggshells was ground using a mortar and pestle, followed by calcination at 900 °C for 3 h. During this process, calcium carbonate (CaCO_3_) was thermally decomposed into calcium oxide (CaO) with the release of carbon dioxide (CO_2_), as shown in Eq. 1

1 $$ {\text{CaCO}}_{{3}} \to {\text{CaO}} + {\text{CO}}_{{2}} \uparrow $$

After calcination, the material was reground and sieved to obtain a fine CaO powder for subsequent use.

#### Synthesis of HAP

HAP was synthesized using calcium oxide (CaO) derived from eggshells through a process of chemical precipitation and calcination. Approximately 1 M of calcined eggshell powder was mixed with 50 mL of deionized water to produce calcium hydroxide [Ca(OH)_2_], as illustrated in Eq. 2.

2 $$ {\text{CaO}} + {\text{H}}_{{2}} {\text{O}} {\text{Ca}}\left( {{\text{OH}}} \right)_{{2}} $$

The chemical precipitation was then carried out by reacting 1 M Ca(OH)_2_ solution with 0.6 M orthophosphoric acid (H_3_PO_4_), as shown in Eq. 3, referring to Pu'ad et al. (2021).

3 $$ {1}0{\text{Ca}}\left( {{\text{OH}}} \right)_{{2}} + {\text{6 H}}_{{3}} {\text{PO}}_{{{4} }} {\text{Ca}}_{{{1}0}} \left( {{\text{PO}}_{{4}} } \right)_{{6}} \left( {{\text{OH}}} \right)_{{2}} + {\text{18H}}_{{2}} {\text{O}} $$

Prior to the titration process, the pH of the 50 ml Ca(OH)_2_ solution was measured. Subsequently, Orthophosphoric acid was added gradually in a dropwise manner to adjust the pH to final values of 9.5, 10.5, and 11.5. After mixing, the solution was permitted to stand at room temperature for 24 h to facilitate precipitation. It was then stirred magnetically for 30 min and allowed to precipitate again for an additional 24 h.

The precipitate was filtered, rinsed with deionized water, and then dried at 100 °C. The resulting powder was subjected to calcination at 700, 950, and 1200 °C for 1, 2, and 3 h, respectively, in a muffle furnace to produce the final HAP product.

### Characterization of HAP

The crystalline structure of HAP was analysed using X-ray diffraction (XRD) with a Shimadzu Lab XRD-6700. Data were collected using the step-scanning method with a step size of 0.02° over a 2θ range of 20°–80°. The resulting diffraction patterns were compared with the standard reference pattern for HAP (ICDD 00–003-0747). The morphology and elemental composition of the powder were examined using a Hitachi S-3400 N Field Emission Scanning Electron Microscope (FESEM) equipped with an energy-dispersive X-ray spectrometer (EDS) operating at an accelerating voltage of 15 kV. Functional group analysis of the HAP sample was conducted using Fourier-transform infrared spectroscopy (FTIR; Spectrum 100, PerkinElmer), with spectra recorded in the range of 600–4000 cm^−1^.

### Statistical analysis using Design of Experiment

Response Surface Methodology (RSM) combined with Central Composite Design (CCD) was used to investigate the synergistic effects of three independent variables: precipitation pH (9.5–11.5), calcination temperature (700–1200 °C), and calcination time (1–3 h), aimed at achieving stoichiometric hydroxyapatite (HAP).The levels and ranges of these factors were determined based on a comprehensive review of literature, preliminary experimental results, and previous studies (Pu'ad et al. 2021; Vinayagam et al. 2023; Muñoz-Sanchez et al. 2023), ensuring relevance to the intended synthesis conditions. The experimental matrix was generated using Design Expert software (version 13.0.15), which was also used to build and evaluate the statistical models and to generate three-dimensional response surface plots.

### Mixed Culture and fermentation medium

The mixed bacterial culture used in this study was acquired from Jamali et al. (2017) and acclimatized in a bioreactor at the Green Technology Laboratory, UPM, to maintain the dominance of *Thermoanaerobacterium Thermosaccharolyticum*-sp. A synthetic fermentation medium was used to support the growth of the mixed culture. The medium contained (per litre of deionized water): NH_4_Cl (1 g), NaCl (2 g), MgCl_2_.6H_2_O (0.5 g), CaCl_2_.2H_2_O (0.05 g), K_2_HPO_4_.3H_2_O (1.5 g), KH_2_PO_4_ (0.75 g), NaHCO_3_ (2.6 g), and yeast extract (2 g). An equal amount of Glucose (5 g/L) and Xylose (5 g/L) were added at ratio 1:1 (v/v) as carbon and energy sources to produce biohydrogen. The fermentation medium was adjusted to pH 6 and incubated at 60 °C in a water bath shaker for 48 h at a rotational speed of 150 rpm (Jamali et al. 2017).

### Design for batch fermentation

Batch experiments were conducted to study BioH_2_ production in Scott duran bottles with a 200 mL working volume. Six different concentrations of HAP (80, 240, 400, 560, 720, and 880 mg/L) were tested to study bioH_2_ production, alongside a control group without HAP. Glucose and xylose (5 g/L each) were added to the reactors as carbon sources. After that, the reactors were flushed with nitrogen gas for 5 min to create an anaerobic environment. Before the experiment, the pH of the medium was adjusted to 6.0 ± 0.3. The fermentation was carried out under thermophilic conditions at 60 °C, following the method described in Jamali et al. 2019b. Samples were collected every 4 h, and cumulative hydrogen production (CHP) was measured using an inverted measuring cylinder. The schematic setup of the thermophilic dark fermentation reactor is provided in the Supplementary Material (Fig. S1).

### Analysis of Biohydrogen Production

The volume of biogas was measured every 4 h for a total duration of 72 h. The hydrogen content in the biogas was analysed using a Gas Chromatograph (Agilent 6980 N, Agilent Technologies, USA) equipped with a capillary column (Agilent 19095P-Q40, 30 m × 530 µm × 40 µm) and a thermal conductivity detector (TCD; Agilent, USA) operated at an injection temperature of 110 °C. Helium was used as the carrier gas at a flow rate of 25 mL/min and a column oven temperature of 55 °C.

To estimate the cumulative hydrogen production and evaluate fermentation kinetics, a modified Gompertz model was applied. In theory, the modified Gompertz equation can be expressed as Eq. 4 follows (Jamali et al. 2021).

4 $${H}_{t}={H}_{m}.exp\left\{-exp\left[\frac{{R}_{m}. e}{{H}_{m}}\left(\left(\lambda -t\right)+1\right)\right]\right\}$$

where, ${H}_{t}$ = cumulative hydrogen production (mL), ${H}_{m}$ = maximum hydrogen production (mL), ${R}_{m}$ = maximum hydrogen production rate (ml/h), e = Euler number, λ = lag phase time (h) and t = incubation time (h).

### Determination of sugar concentration

The samples were filtered through a 0.22 µm syringe filter and collected in vials. Sugar and soluble volatile fatty acid (VFA) concentrations were quantified using an Agilent 1200 HPLC system (California, USA), equipped with a REZEX ROA column (Phenomenex, USA) and a refractive index detector (RID). The mobile phase was 5 mM H_2_SO_4_, operated at a constant flow rate of 0.6 mL/min and a column temperature of 25 °C. All analyses were conducted in triplicate, and results are expressed as mean ± standard deviation (SD).

### Microbial community analysis

Microbial community analysis was conducted to identify the microorganisms involved in the fermentation process. Cells were harvested by centrifugation at 7000 rpm for 10 min (Jamaludin et al. 2023). and genomic DNA was extracted using the FastDNA™ Spin Soil Kit (MP Biomedicals). The quality of the extracted DNA was assessed using 1% TAE agarose gel electrophoresis. Amplification of the 16S rRNA gene was performed using primers specific to the V3–V4 hypervariable regions. The forward primer was CCTACGGGNGGCWGCAG, and the reverse primer was GACTACHVGGGTATCTAATC. PCR reactions were carried out using REDiant® 2X PCR Master Mix (1st BASE). To enable sequencing, overhang adapters were added:

Forward: TCGTCGGCAGCGTCAGATGTGTATAAGAGACAG.

Reverse: GTCTCGTGGGCTCGGAGATGTGTATAAGAGACAG.

Amplicons were further indexed using the Nextera® XT Index Kit v2, following Illumina’s instructions. The final library quality was validated using the Agilent Bioanalyzer 2100 System with the DNA 1000 Kit and quantified using Helixyte® Green™ fluorometric reagent. Sequencing was performed on the Illumina MiSeq platform using paired-end 300 bp reads. The obtained sequences were analyzed using the SILVA NR reference database (version 138.1). Taxonomic classification was carried out using BLAST searches against GenBank sequences (Jamali et al. 2016).

## Results and Discussion

### Statistical process optimization of HAP stoichiometry using response surface methodology (RSM)

A Response Surface Methodology (RSM) was utilised to evaluate the most significant interaction between key parameters for producing stoichiometric HAP. A central composite design was employed to develop a system model that identifies the feasible interactions among these parameters within a minimal number of experimental runs. Table 1 displays CCD of three parameters, with the HAP Ca/P ratio as the response. The desired response Ca/P value 1.67 was found at the centre point of the design, corresponding to a pH of 10.5, a calcination temperature of 950 °C, and a duration of 2 h.

**Table 1:** The result of RSM-based experimental optimization

Run	Factor A	Factor B	Factor C	Response
	pH	Temp. (℃)	Time (h)	Ca/P (EDS) Ratio
1	9.5	700	1	1.48
2	11.5	700	1	1.68
3	9.5	1200	1	1.64
4	11.5	1200	1	1.73
5	9.5	700	3	1.49
6	11.5	700	3	1.62
7	9.5	1200	3	1.62
8	11.5	1200	3	1.72
9	9.5	950	2	1.6
10	11.5	950	2	1.72
11	10.5	700	2	1.57
12	10.5	1200	2	1.66
13	10.5	950	1	1.65
14	10.5	950	3	1.7
15	10.5	950	2	1.67
16	10.5	950	2	1.67
17	10.5	950	2	1.67
18	10.5	950	2	1.67
19	10.5	950	2	1.67
20	10.5	950	2	1.67

**Fig. 1:**
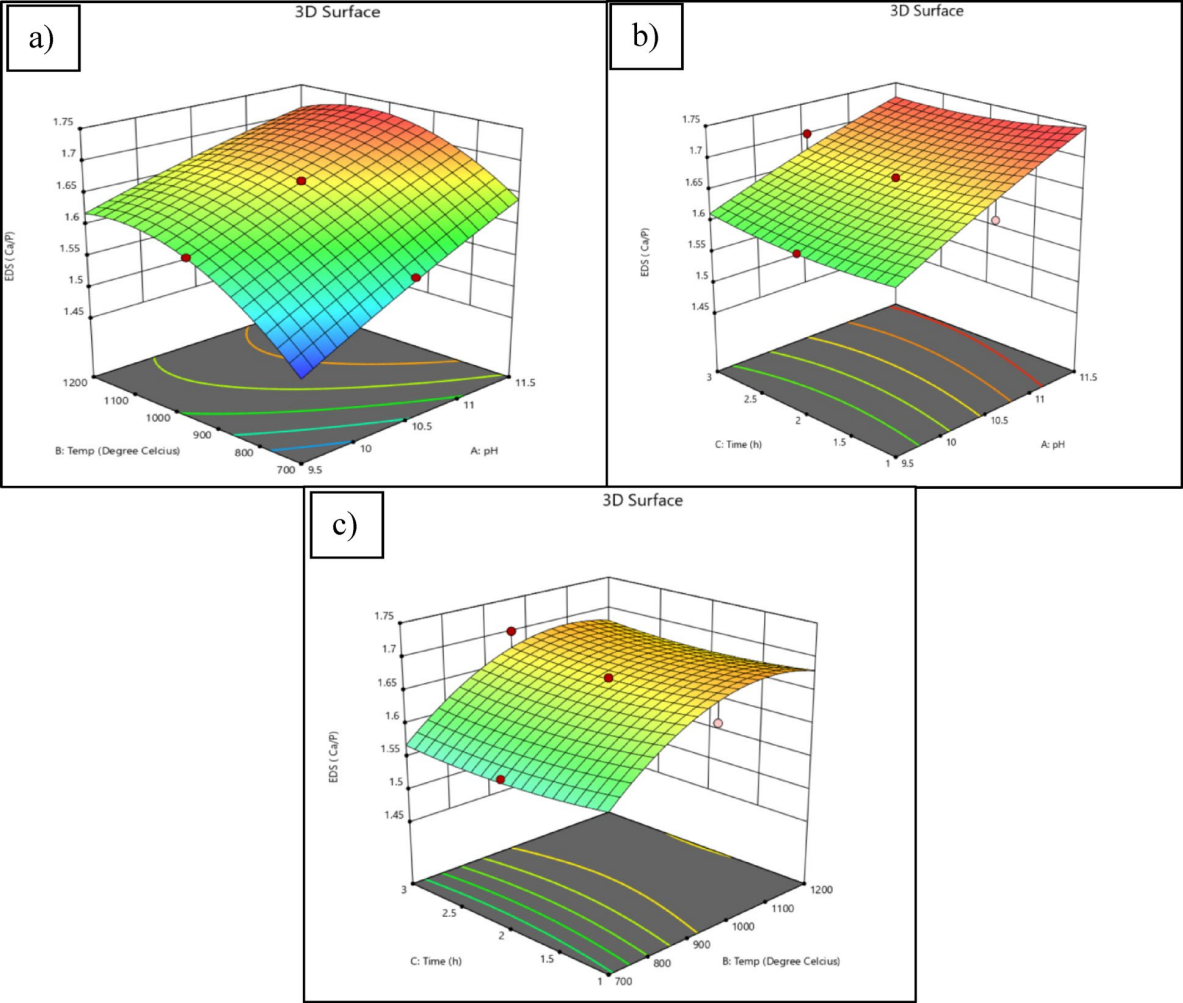
3D-Surface Model Graph illustrating the interaction effects of **a** pH and calcination temperature (A and B), **b** pH and calcination time (A and C), and **c** calcination temperature and time (B and C) on the Ca/P ratio

The interaction effects of the three variables on the Ca/P ratio were visualized using 3D surface response plots, as shown in Fig. 1. Each plot represents a pairwise interaction between two variables, while the third variable was held constant at the centre point (zero level).

In Fig. 1a, a clear interaction is observed between precipitation pH (Factor A) and calcination temperature (Factor B), with the Ca/P ratio peaking near the centre region. This indicates a synergistic effect between these two parameters in achieving stoichiometric HAP. Optimal synthesis conditions were pH 10.5, a calcination temperature of 950 °C, and a duration of 2 h, which resulted in a Ca/P ratio of 1.67. This ratio is consistent with the theoretical value for HAP and is supported by previous studies (Pu'ad et al. 2021; Vinayagam et al. 2023; Ferro and Guedes 2019; Muthu et al. 2022).

Figures 1b and 1c highlight weaker interactions involving calcination time (Factor C). A data point located beneath the response surface in each graph—specifically at (10.5, 1, 1.65) for Fig. 1b and (950, 1, 1.65) for Fig. 1c—indicates that calcination time had a minimal effect on the Ca/P ratio when compared to pH and temperature. This suggests that variations in time within the studied range were not significantly correlated with response changes (Xia and Wang 2022).

The findings further reinforce the importance of maintaining a pH ≥ 10 for successful HAP formation, as this condition promotes both nucleation and crystallization (Lala et al. 2021). Although prior studies have noted that HAP can begin forming above pH 4.2 (Goh et al. 2021), the current results validate that a pH of 10.5 is optimal for producing stoichiometric particles (Ibrahim et al. 2015).

The calcination temperature of 950 °C also emerged as a critical parameter, yielding a Ca/P ratio close to 1.67. Atomic force microscopy (AFM) analyses in related literature confirm that such temperatures support the formation of nano- to microscale HAP crystals (Hammood et al. 2019). Furthermore, when bio-waste materials were calcined at 950°C, they produced micro-HAP similar to commercial nano-HAP (Ramirez-Gutierrez et al. 2017). On the other hand, it was reported that HAP synthesised from the poultry eggshells at 1000 ℃ of calcination temperature produces a slightly lower Ca/P ratio of 1.65, similar to natural bone composition (Agbabiaka et al. 2020).

### Statistical analysis (ANOVA)

Response Surface Methodology (RSM) employing a Central Composite Design (CCD) was used to assess the interactive effects of three factors—pH, calcination temperature, and calcination time—on the Ca/P ratio of synthesized HAP. A second-order quadratic model was applied to the experimental data, and its adequacy was validated using Analysis of Variance (ANOVA).

The regression equation derived from the model is expressed as follows:

5 $$ \begin{aligned} {\text{Ca}}/{\text{P}}\;{\text{ratio}}\;\left( {{\text{EDS}}} \right) & = {1}.{63} + 0.0{\text{695A}} + 0.0{\text{617B}} + 0.000{\text{8C}} \\ & - 0.0{175}\left( {{\text{AB}}} \right) - 0.00{75}\left( {{\text{AC}}} \right) + 0.00{25}\left( {{\text{BC}}} \right) \\ & - 0.00{55}\left( {{\text{A}}^{{2}} } \right) - 0.0{5}0{5}\left( {{\text{B}}^{{2}} } \right) + 0.00{95}\left( {{\text{C}}^{{2}} } \right) \\ \end{aligned} $$

where A = pH, B = Temperature and C = Calcination time.

The ANOVA results in Table 2 confirm the model’s significance, with a high F-value (30.77) and a p-value < 0.0001. Among the model terms, pH (A) and temperature (B) showed highly significant effects (p < 0.0001), while calcination time (C) was found to be statistically insignificant (p = 0.5976). Significant two-way interactions were observed between pH and temperature (AB), whereas other interaction and squared terms had minimal effects.

**Table 2:** ANOVA for RSM parameter

Source	Sum of Squares	df	Mean Square	F-value	P-value	
Model	0.0839	9	0.0093	30.77	< 0.0001	Significant
A-pH	0.0410	1	0.0410	135.26	< 0.0001	
B-Temp	0.0281	1	0.0281	92.76	< 0.0001	
C-Time	0.0001	1	0.0001	0.2972	0.5976	
AB	0.0025	1	0.0025	8.09	0.0174	
AC	0.0004	1	0.0004	1.49	0.2508	
BC	0.0001	1	0.0001	0.1651	0.6930	
A^2^	0.0001	1	0.0001	0.2702	0.6145	
B^2^	0.0070	1	0.0070	23.12	0.0007	
C^2^	0.0003	1	0.0003	0.8275	0.3844	
Residual	0.0030	10	0.0003			
Lack of Fit	0.0030	5	0.0006			
Pure Error	0.0000	5	0.0000			
Cor Total	0.0869	19				

The ANOVA analysis results for the response parameter can be observed in Table 3. The R^2^ value denotes the proportion of the measured response value variation that can be clarified through the experimental variables and their relationship to each other. The coefficient of determination (R^2^) varies from 0 to 1, with higher values signifying a more robust model and better response prediction (Rafie and Nordin 2017). The R^2^ value in this test is 0.9652, indicating a robust model that explains a substantial amount of the discrepancy in the measured response value.

**Table 3:** ANOVA result for response parameter

Std. Dev	0.0174	R^2^	0.9652
Mean	1.65	Adjusted R^2^	0.9338
C.V. %	1.06	Predicted R^2^	0.5886
		Adeq Precision	19.8296

The analysis confirms that pH and temperature are the most influential parameters in optimizing the Ca/P ratio. Although calcination time was part of the design, its high p-value and low F-value indicate that it had no statistically significant effect on the stoichiometry of the final HAP product. This result aligns with previous studies that reported successful synthesis of HAP at various calcination durations. For example, HAP has been synthesized at 1100 °C for 1 h (Ćurković et al. 2017), and also at 900 °C and 700 °C for 2 h in other studies (Muñoz-Sanchez et al. 2023). Another study reported synthesis at 950 °C for 4 h (Ibrahim et al. 2015). These results suggest that while the duration of calcination can influence crystal growth or particle size, it does not significantly impact the Ca/P ratio. Therefore, calcination time was held constant at 2 h for subsequent experiments, as supported by the surface plots (Fig. 1b).

**Fig. 2:**
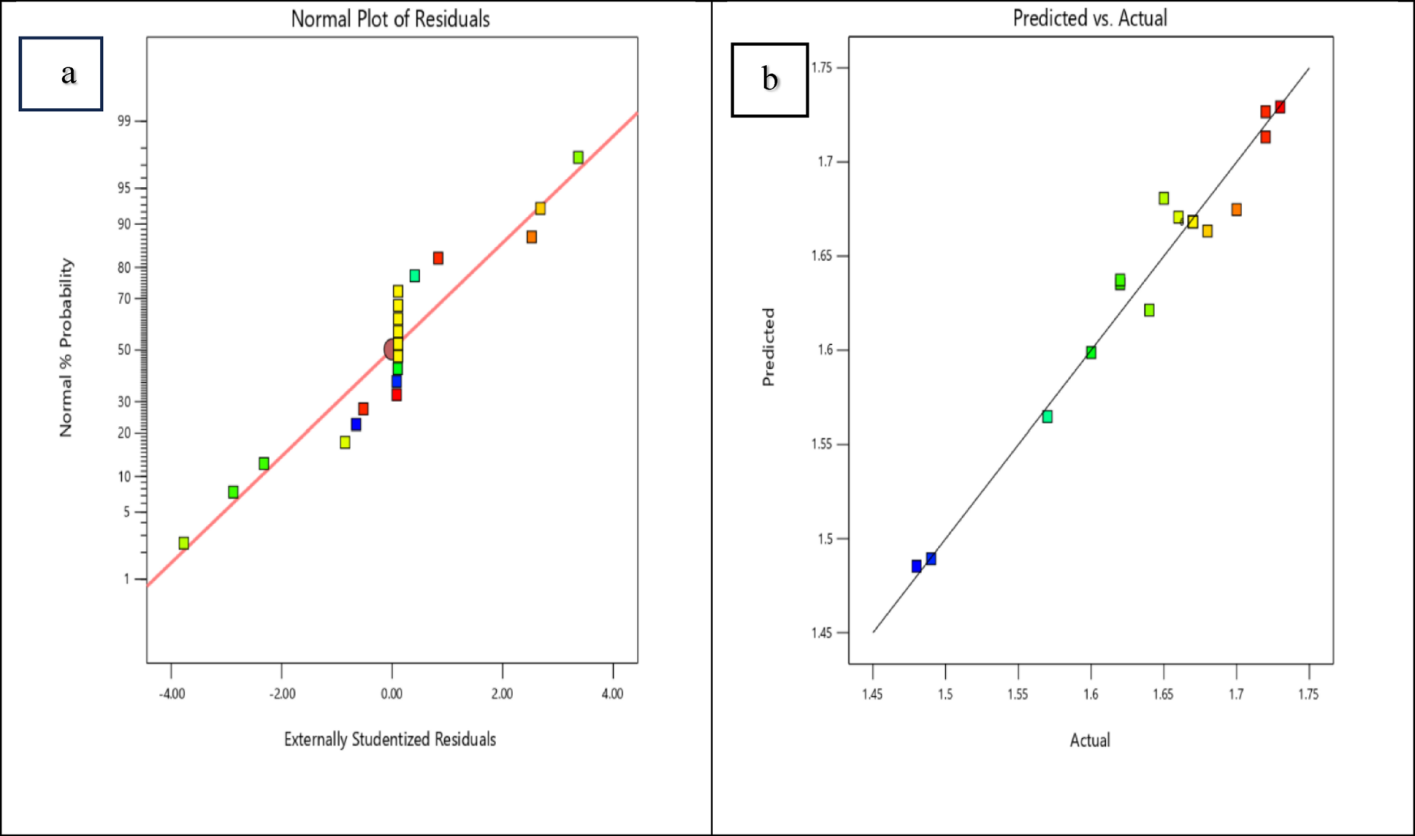
Residual diagnostic plots: **a** Normal probability plot of externally studentized residuals, and **b** Predicted versus actual response plot for Ca/P ratio of HAP

To evaluate the reliability of ANOVA, it is mandatory to perform residual analysis, in addition to achieving a satisfactory R^2^ value. Figure 2 displays two selected influencing plots. The plot of normally distributed versus residuals indicates that all residuals were evenly scattered along diagonal line (Fig. 2a). If there is a "S-shaped" curve, a modification should be used. The scatter plot in Fig. 2b validates that the projected data had a random distribution around their corresponding actual values, with no discernible patterns. This phenomenon demonstrates that the proposed model was suitable, and any deviations from the assumptions of independence or constant variance can be rejected.

### EDS Characterization of HAP

The optimized HAP sample obtained under conditions identified by RSM (Ca/P ratio = 1.67) was further characterized using Energy Dispersive X-ray Spectroscopy (EDS), in conjunction with XRD, FTIR, and FESEM analysis.

EDS analysis (Fig. 6d) revealed the presence of Ca, P, and O as the major elemental components of the sample, while trace amounts of C, Mg, and Al were also detected. These minor elements likely originated from the eggshell precursors and are consistent with those typically observed in natural bone matrices (Goh et al. 2021). The lack of additional impurity peaks indicates the high purity of the prepared HAP, in line with previous studies (Kamieniak et al. 2016). The atomic Ca/P ratios were calculated from the EDS results (Table 1), showing an average value of 1.67, which corresponds well with the stoichiometric ratio for HAP and corroborates the XRD findings.

Specifically, the sample calcined at 950 °C for 2 h at pH 10.5 exhibited a Ca/P ratio of 1.67, confirming the formation of stoichiometric HAP. This agreement between EDS and XRD further validates that the sample predominantly comprises a crystalline hydroxyapatite phase. All samples showed Ca/P ratios ranging from 1.48 to 1.72, which is within the range of stoichiometric HAP (1.67) and β-TCP (1.43). These findings suggest that when calcined under optimal conditions, the eggshell-derived precursors yielded high-purity hydroxyapatite. Samples calcined at 950 °C, pH 10.5, and for 2 h were found to contain only HAP phases, as confirmed by the combined EDS, XRD, and FTIR data.

The Ca/P ratio of the raw eggshells also fell within the range of 1.48–1.72, aligning with the reported values for calcium-deficient hydroxyapatite (CDHA). This further supports the idea that eggshells are an effective source of calcium for HAP synthesis. When Ca/P > 1.67, calcium oxide (CaO) tends to form upon calcination. In contrast, a Ca/P < 1.67 favors the formation of β-TCP (Zhu et al. 2017). Therefore, maintaining the Ca/P ratio near 1.67 is critical for directing the phase composition toward stoichiometric HAP.

### Powder X-ray diffraction analysis

**Fig. 3:**
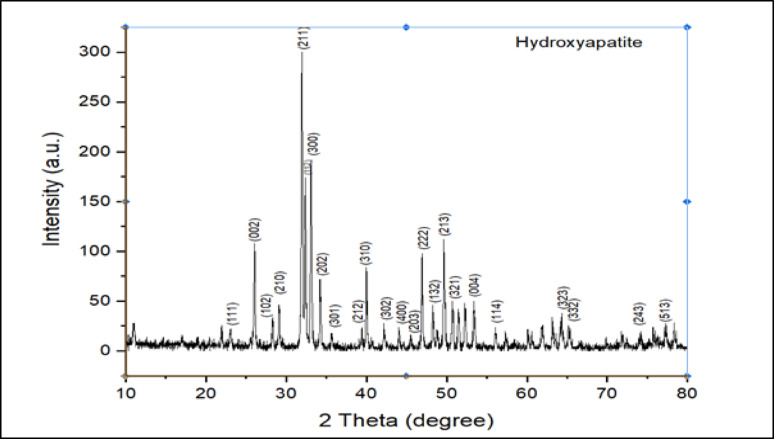
XRD pattern of the optimized HAP powder sample calcined at 950 °C

The XRD pattern of the optimized HAP sample calcined at 950 °C (Fig. 3) indicates a polycrystalline structure with well-defined peaks, showing a preferred orientation along the (211) plane. Major diffraction peaks were observed at 2θ values of 25.79°, 28.88°, 29.54°, 31.16°, 32.05°, 32.51°, 34.45°, 35.27°, 38.57°, 40.05°, 48.56°, 49.13°, and 53.03°. These reflections were indexed to the (002), (102), (210), (211), (112), (300), (202), (301), (212), (310), (213), and (004) planes, consistent with standard hydroxyapatite data (JCPDS No. 09–0432) (Selimin et al. 2022). These results confirm the formation of phase-pure HAP.

The sharp peak at 31.98°, corresponding to the (211) plane, further supports a preferred crystallographic orientation in this sample. Similar findings have been reported for HAP synthesized using plant-based bio-templates such as *Peltophorum pterocarpum* (Sodhani et al. 2022).

The sample's average crystallite size was determined using the Debye–Scherrer equation, as shown in Eq. 6 (Wijesinghe et al. 2014).

6 $$ D = 0.93\lambda /\beta \cos \theta $$

Here, λ represents the X-ray wavelength of Cu Kα radiation, which is 1.54 Å. β indicates the full width at half maximum of the principal XRD peak, while θ denotes the angle of diffraction. Using this equation, the estimated crystallite size was 44.91 nm, suggesting nanoscale crystal domains.

The degree of crystallinity was evaluated using Eq. 7

7 $$ {\text{Crystallinity}}\;\left( \% \right) = \left( {{\text{B}}/\left( {{\text{A}} = {\text{B}} + {\text{C}}} \right)} \right) \times 100 $$

where B = the area of all crystalline peaks, A = the area of all peaks and C = the area of the amorphous peaks. By utilizing this equation and XRD data depicted in Fig. 3, the crystallinity of the HAP sample was calculated to be approximately 78%. This value is consistent with the results reported by Karampour et al. (2022), who obtained a crystallinity of 71% for HAP synthesized under similar conditions. Therefore, it can be concluded that eggshell waste can serve as a natural source for producing highly crystalline green HAP particles.

### FTIR Spectra of the HAP

**Fig. 4:**
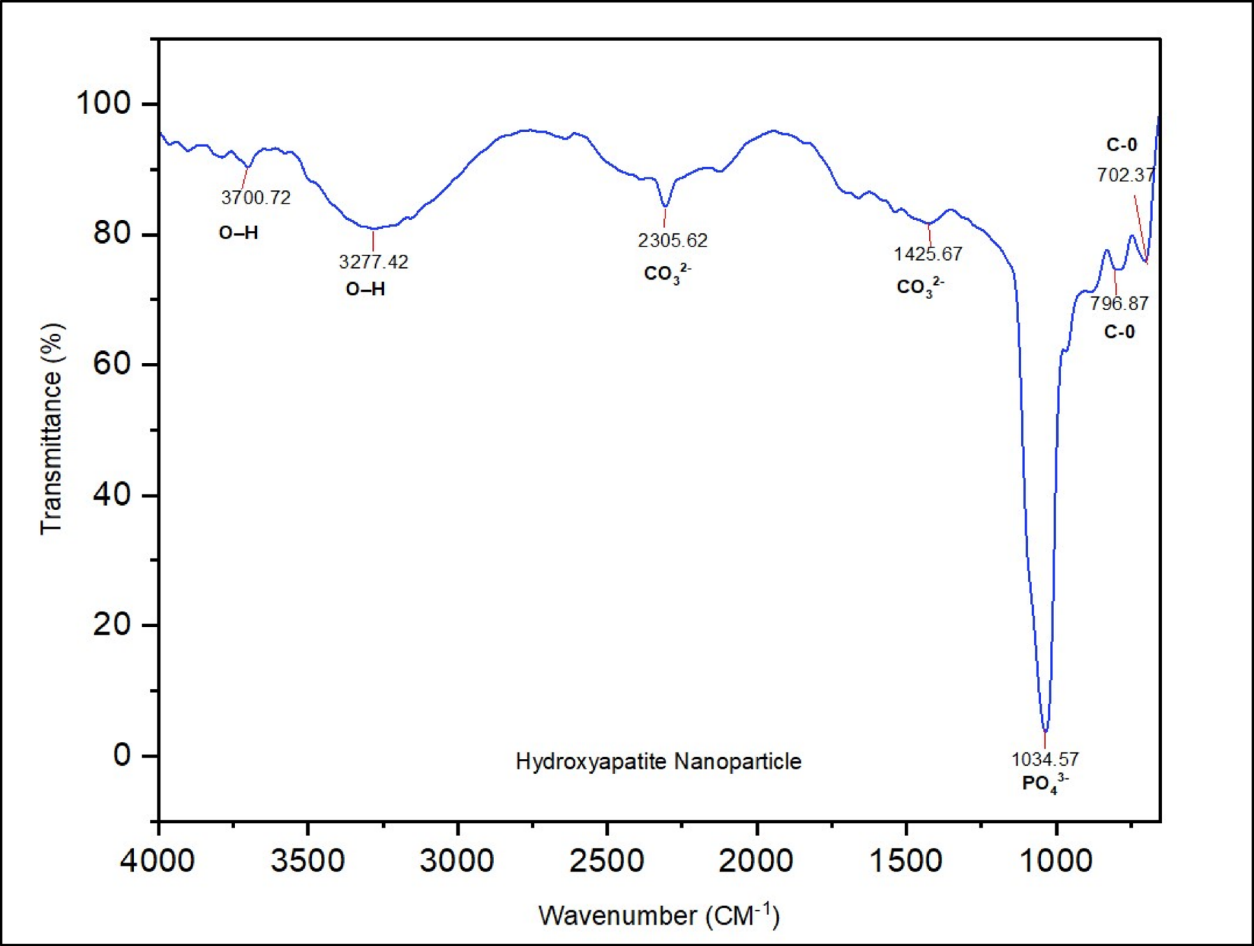
The FTIR spectra of HAP

The FTIR spectrum of the synthesized hydroxyapatite (HAP) in the range of 650–4000 cm^−1^ is shown in Fig. 4. Several distinct absorption bands confirm the functional groups associated with the HAP structure. A strong and sharp peak observed at 1034.57 cm^−1^ corresponds to the antisymmetric stretching vibration (ν_3_) of phosphate groups (PO_4_^3−^), indicating the presence of the HAP phase (Adeogun et al. 2018). Additional phosphate-related peaks within the 900–1066 cm^−1^ region are attributed to symmetric (ν₁) and antisymmetric (ν_3_) P–O stretching vibrations (Sinusaite et al. 2019; Gu et al. 2013), further validating the phosphate framework of HAP. Characteristic peaks confirmed hydroxyl groups (OH^−^) at 3700.72 cm^−1^ and 3277.42 cm^−1^, which correspond to O–H stretching and bending vibrations, respectively. The presence of sharp O–H peaks is indicative of well-crystallized hydroxyapatite and confirms structural integrity (Umesh et al. 2021). Carbonate impurities (CO_3_^2−^) were identified at 2305.62, 1425.67, 796.87, and 702.37 cm^−1^. The relatively low intensity of these bands suggests the presence of CO_3_^2−^ in trace quantities. The peak at 1425.67 cm^−1^ corresponds to the ν_3_ asymmetric stretching vibration of CO_3_^2−^, indicating A-type substitution, where carbonate ions replace hydroxyl groups in the HAP lattice (Lala et al. 2021). The minor intensity of the ν_2_ bands at 796.87 and 702.37 cm^−1^ also supports the low concentration of carbonate (Pu'ad et al. 2021).

Together, these spectral features confirm the formation of A-type HAP with high structural purity and crystallinity, consistent with previous structural characterization results.

### FESEM Morphology studies of HAP

**Fig. 5:**
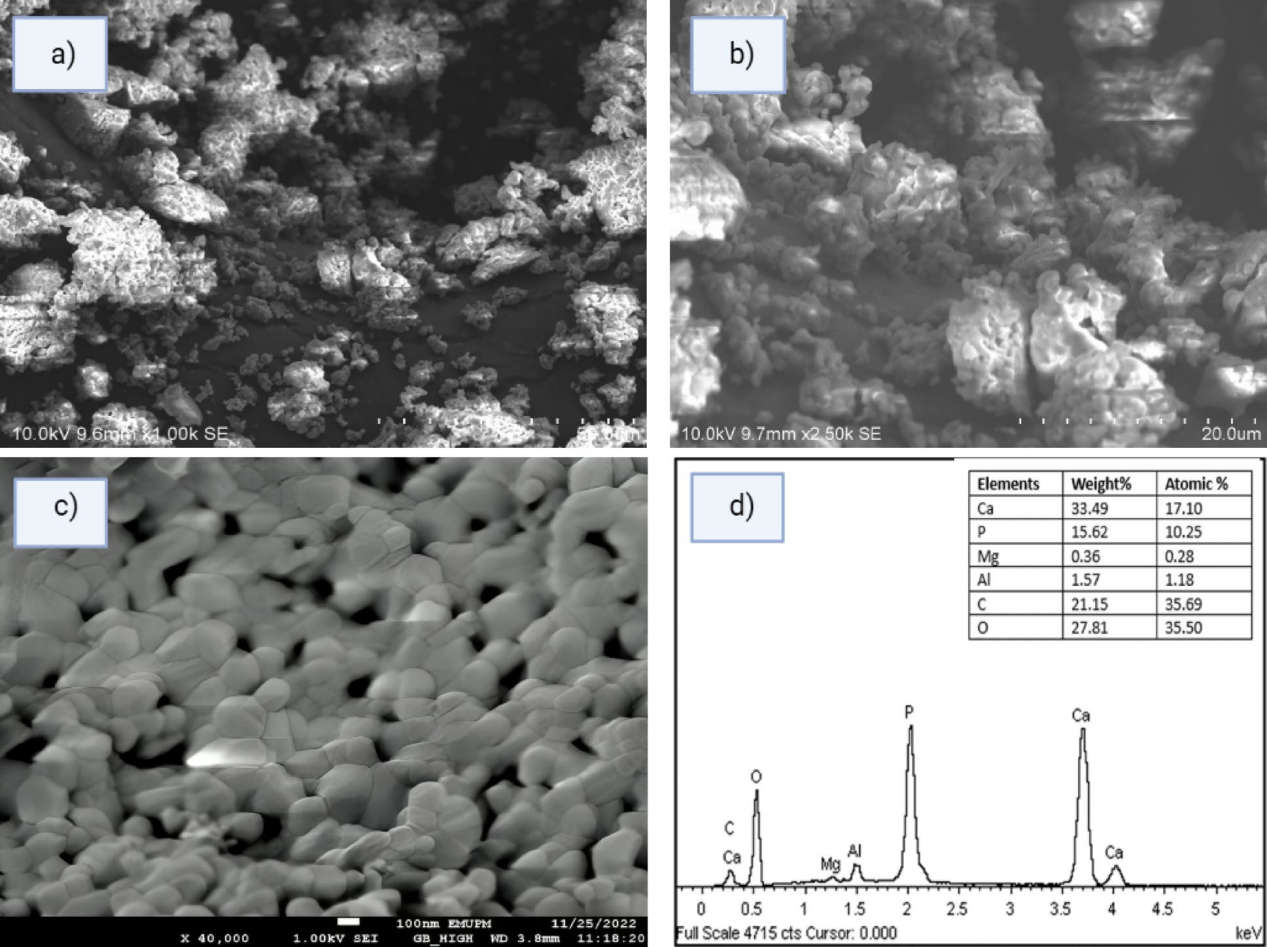
FESEM and EDS analysis of HAP synthesized at pH 10.5, 950 °C, 2 h: (**a**) 1000×, (**b**) 2500×, (**c**) 10,000× FESEM images, and (**d**) EDS spectrum and elemental composition (Ca/P = 1.67)

The morphology of the synthesized hydroxyapatite (HAP) was analyzed using Field Emission Scanning Electron Microscopy (FESEM), as shown in Fig. 5(a–c). The micrographs reveal agglomerated, near-spherical particles with an average size of ~ 100 nm. At higher magnifications, the particles exhibit interconnected porous structures with surface irregularities, which are known to increase the specific surface area—a desirable property for applications involving adsorption and biological interactions (Mobika et al. 2020; Abidi and Murtaza 2014).

Elemental composition was confirmed by Energy-Dispersive X-ray Spectroscopy (EDX) (Fig. 5d), which detected Ca, P, and O as the major elements. The Ca/P atomic ratio was 1.67, consistent with stoichiometric hydroxyapatite. Minor elements such as C, Mg, and Al originated from the precursor material (Vinayagam et al. 2023; Mo et al. 2022).

### BioH_2_ yield affected by bio-additive of HAP

**Fig. 6:**
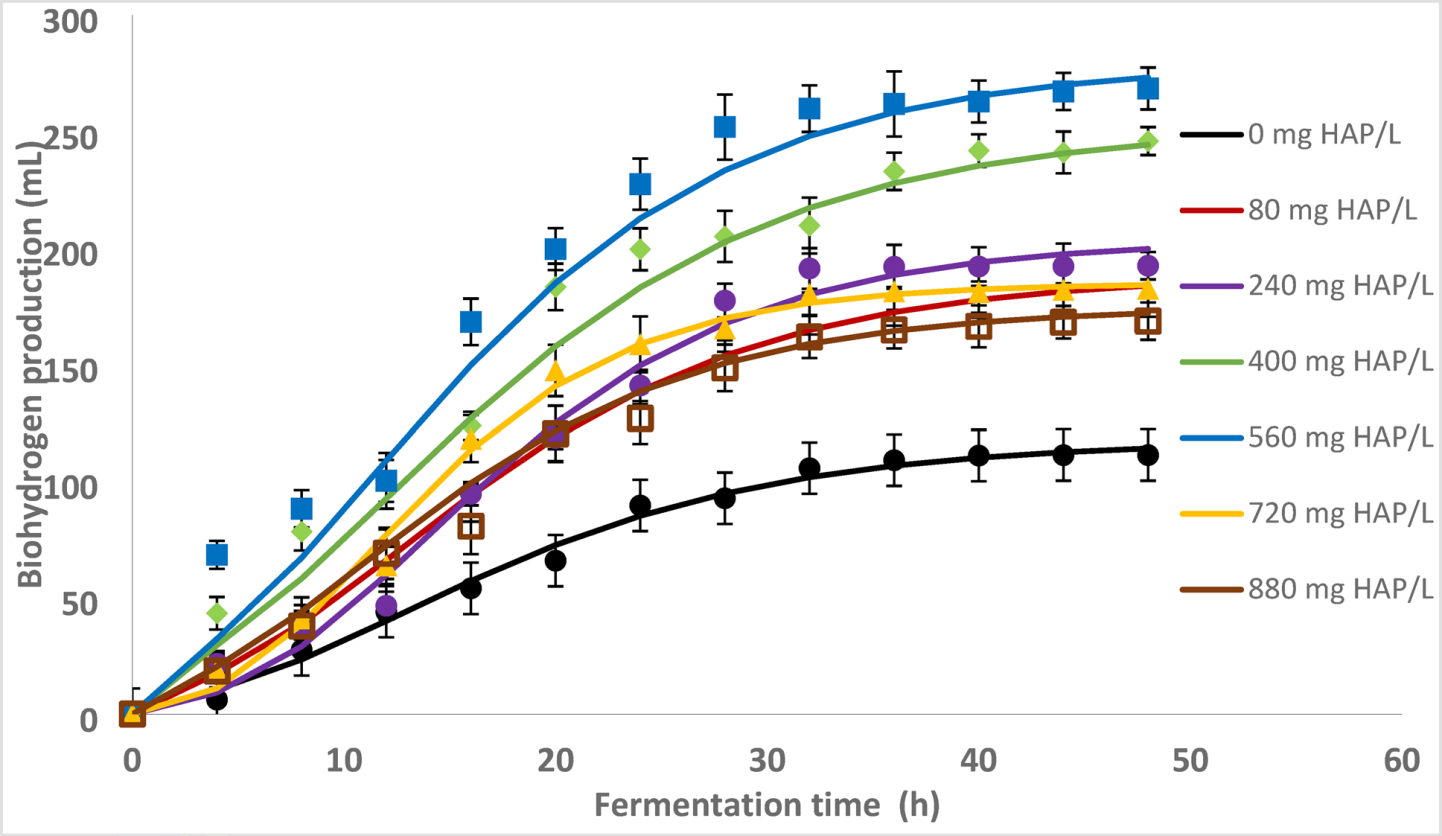
Gompertz curve-fitting graph of biohydrogen production with different dosages of HAP

The effect of hydroxyapatite (HAP) as a bio-additive was investigated under batch thermophilic conditions using different concentrations (0–880 mg/L). As shown in Table 4, the addition of HAP significantly enhanced hydrogen production compared to the control (0 mg/L). The maximum hydrogen volume (268.5 mL) and yiel (1.085 mol H_2_/mol sugar) were achieved at 560 mg/L HAP, with a hydrogen content of 51.32 ± 1.54% (Fig. 6). Beyond this concentration, the performance declined, indicating an optimal threshold. The hydrogen production rate (HPR) and yield (HY) increased with rising HAP concentration up to 560 mg/L, after which a decreasing trend was observed. This aligns with previous studies showing enhanced bioH_2_ production with moderate levels of calcium-based additives such as CaO and CaO_2_ (Lay et al. 2020; Aziz et al. 2022; Zainal et al. 2020). As summarized in Table 5, variations in additive type, synthesis method, substrate, and microbial community significantly influence the performance.

**Table 4:** The effects of Hydroxyapatite on thermophilic bacteria

Concentration (mg HAp/L)	Vol H_2_ (mL)	Sugar utilization %(v/v)	Final pH	H_2_ Production Rate ml H_2_/(L.h)	Yield (mol H_2_/mol sugar)	Modified Gompertz equation parameter values for H_2_ production			Biomass (g VSS/L)
						H_m_ (mL)	R_m_ (mL/h)	λ (h)	
0	111.22	93.6	93.6	11.585	0.437	117.36	4.36	2.82	1.24
80	178.42	91.8	91.8	18.585	0.715	188.76	7.09	2.68	1.60
240	192.38	92.1	92.1	20.039	0.768	203.49	8.63	5.09	1.98
400	235.9	91.7	91.7	24.573	0.946	252.52	8.81	1.5	2.51
560	268.5	91.0	91.0	27.969	1.085	279.54	10.63	1.76	2.86
720	182.5	92.0	92.0	19.011	0.729	184.96	9.92	4.22	1.96
880	168.58	91.5	91.5	17.560	0.678	174.78	7.15	1.89	1.83

**Table 5:** Studies on the impact of hydroxyapatite in biohydrogen production

No	Immobilizer	Synthesis Method	Surface area (m^2^/g)	Working Volume (mL)	Temperature (ᵒC)	Optimum Concentration	Culture	Substrate	Yield	References
1	HAP	coprecipitation	4.751	500	37	400 mg/L HAP	Mixed Culture	Glucose	182.33 ± 2.41 mL/g glucose	Mo et al. 2022)
2	Graphene/hydroxyapatite nano-composite (nG/HAP)	hydrothermal method	-	200	35 ± 0.2	80 mg/gVS nG/HAP	Mixed Culture	delignified duckweed (DDW)	208.9 ± 12.6 mL/g DDW	Tawfik et al. 2022)
3	Graphene/hydroxyapatite nanoparticles (GHN)	hydrothermal method	-	250	35	50 mg/gVS	Mixed culture	black liquor	2.187 mol/mol glucose	Tawfik et al. 2021)
4	HAP	Green Synthesis (From eggshell)	4.887	200	60	560 mg/L HAP	Mixed culture	POME	1.085 mol H_2_/mol sugar	This study

The improved hydrogen production noted in this study is directly related to the physicochemical properties of the synthesized HAP derived from eggshells. The XRD and FTIR analyses confirmed that the structure is highly crystalline and stoichiometric, with a calcium-to-phosphorus ratio of approximately 1.67. This composition facilitates controlled dissolution and the gradual release of calcium ions (Ca^2^⁺) into the fermentation medium, which aligns with findings from previous studies on calcium-based additives (Elfaki et al. 2024; Luo et al. 2024). This gradual ion release prevents sudden pH spikes, maintaining a stable microenvironment that is favorable for the growth and metabolic activity of *Thermoanaerobacterium* (Zhang et al. 2022). The FESEM images showed a porous surface morphology that enhances microbial adhesion and mass transfer, similar to what was reported for HAP-assisted granulation (Xue et al. 2022). Thus, the combination of crystallinity, biocompatibility, and a porous texture in eggshell-derived HAP enhances buffering and microbial stability and promotes sustained hydrogen evolution under thermophilic conditions. The structural and compositional factors explain the increased hydrogen yield and stability at optimal HAP loading compared to the control system.

Based on the findings of this investigation, sugar consumption was elevated in all of the samples, exceeding 90%. This suggests that the mixed culture could effectively use the substrate under the experimental conditions. The control group with 0 mg HAP/L exhibits the lowest final substrate concentration, resulting in the highest substrate consumption percentage. This is followed by 240 mg HAP, 720 mg HAP /L, 80 mg HAP /L, 400 mg HAP /L, 880 mg HAP /L, and lastly 560 mg HAP /L. The results indicate that hydroxyapatite with lower concentrations exhibits greater substrate absorption activity than hydroxyapatite with higher concentrations. Prior research has observed that the limited utilization of the substrate may be attributed to the buildup of liquid fermentation, resulting in excessive acidity of the cultures. As a consequence, bacteria are only able to convert a small portion of the substrate due to rate-limiting phases in the process (Malik et al. 2014). The controlled release of Ca^2^⁺ from HAP plays a vital role in stabilizing pH, supporting microbial activity, and promoting favorable metabolic pathways (Haider et al. 2017; Joshi et al. 2018; Wu et al. 2016). Unlike CaO, which releases Ca^2^⁺ rapidly and causes sharp pH increases, HAP releases Ca^2^⁺ gradually, maintaining a stable environment (Elfaki et al. 2024; Luo et al. 2024; Mullai et al. 2013). This sustained ion release enhances microbial performance and supports biohydrogen production over extended periods (Gadhe et al. 2015).

Several studies support the effectiveness of calcium-based additives in enhancing biohydrogen production. The maximum yield of 1.085 mol H_2_/mol sugar at 560 mg/L in this study was 59.73% higher than the control (0.437 mol H_2_/mol sugar), with an HPR improvement of 53.81%. Similar enhancements have been reported for CaO and CaO_2_ (Mo et al. 2022; Zhang et al. 2020). In particular, CaO_2_ has improved short-chain fatty acid production and sludge stability in waste-activated systems (Liang et al. 2022). HAP offers additional advantages, including promotion of microbial granulation and retention. Although HAP concentrations below 400 mg/L were previously reported to be effective (Mo et al. 2022), others have observed the highest H_2_ yield at 1000 mg/L of Ca^2^⁺ (Sekoai and Daramola 2018). These variations are attributed to differences in additive properties, microbial communities, and operating conditions. HAP also enhances microbial aggregation due to its high affinity for bacterial cells (Sun et al. 2021a). However, excessive levels of additives might cause oxidative stress and cell membrane rupture (Wang and Chen 2016). As a result, higher HAP concentrations (over 560 mg/L) might cause granular sludge to lose its structure and make the environment unfavorable for the formation of bioH_2_ (Mo et al. 2022). While these results are consistent with previous findings using Ca-based additives, the novelty of this study lies in the use of green-synthesized HAP from eggshell waste under thermophilic conditions, along with its detailed evaluation of dosage-dependent performance, Ca^2^⁺ release behavior, and impact on microbial biomass. Furthermore, this is among the few studies to simultaneously correlate biohydrogen yield, sugar conversion, and VSS biomass with controlled HAP dosing, providing a comprehensive insight into the mechanistic role of HAP in dark fermentation.

The cumulative hydrogen production profiles at different HAP concentrations were modelled using the modified Gompertz equation, which demonstrated excellent correspondence between experimental and simulated data (R^2^ = 0.975–0.993), confirming its reliability for thermophilic batch fermentation. Both the maximum hydrogen potential (Hm) and production rate (Rm) progressively increased with HAP dosage, rising from 117.36 mL and 4.36 mL h⁻^−1^ in the control group to 279.54 mL and 10.63 mL h^−1^ at a dosage of 560 mg L^−1^. This consistent improvement shows that moderate HAP addition enhanced microbial activity and enzyme efficiency by providing a continuous release of Ca^2^⁺, which buffered the medium and stabilized hydrogenase function. The lag phase (λ) showed a dose-dependent trend, initially decreasing from 2.82 h to 1.76 h as HAP increased to 560 mg L^−1^, indicating faster microbial adaptation and an earlier onset of hydrogenogenesis. At higher concentrations (≥ 720 mg L^−1^), both Hm and Rm declined, while λ increased to 4.22 h. This suggests that excessive Ca^2^⁺ levels induced mild ionic stress or diffusion resistance, which ultimately limited hydrogen evolution.

The shape of the Gompertz curves (Fig. 6) reflects these quantitative trends. At lower HAP concentrations, gas evolution showed a longer adaptation phase followed by a moderate exponential increase. In contrast, at a concentration of 560 mg L^−1^, the curves exhibited a steep rise and reached an earlier plateau, indicating rapid substrate conversion and high metabolic efficiency. At higher additive levels (≥ 720 mg L^−1^), the plateau was reached sooner with lower total H_2_, suggesting either inhibitory stress or diffusion limitations within the medium. Similar kinetic behaviour has been observed in Ca-based and nanoparticle-assisted dark fermentation systems. Optimal additive loading stimulates microbial activation, while excessive dosages induce mass-transfer resistance and redox imbalance (Guo and Wang 2024; Wang and Guo 2024). Overall, the Gompertz analysis offers quantitative evidence that HAP stabilizes the microenvironment, accelerates the exponential phase, and maintains balanced hydrogenase activity, thereby enhancing both the kinetics of hydrogen production and stability of thermophilic dark fermentation.

Although the maximum hydrogen yield achieved in this study (1.085 mol H_2_ mol^−1^ sugar) was slightly lower than some thermophilic reports, this variation stems from fundamental biochemical and operational factors rather than experimental inconsistency. In sealed-batch operation, the gradual buildup of hydrogen in the headspace increases its partial pressure, which thermodynamically suppresses further hydrogenase activity and redirects electrons toward reduced metabolites. The detected volatile fatty acid profile, dominated by butyric and acetic acids, reflects typical electron redistribution through the butyrate–acetate pathway, inherently limiting the stoichiometric yield to about two moles of H_2_ per hexose(Zhang et al. 2022). Mixed-culture thermophilic consortia also include homoacetogens and solventogens that compete for reducing equivalents. This competition enhances process stability but reduces the overall hydrogen fraction (Barth et al. 2024). Furthermore, the yield here was conservatively expressed on a measured-sugar basis without correction for dissolved H_2_, while several comparative studies normalize to total carbohydrate or COD, resulting in numerically higher values (Gadhe et al. 2015). Collectively, these factors explain the slight numerical difference and confirm that the current yield is within a realistic range for batch thermophilic dark-fermentation systems enhanced with calcium-based additives.

Biomass accumulation (g VSS/L) increased with HAP dosage, peaking at 2.86 g/L for 560 mg/L HAP, and followed a trend similar to the hydrogen yield. This indicates that the biohydrogen enhancement was closely associated with bacterial growth. However, at higher concentrations (> 560 mg/L), a decline in both biomass and yield was observed. This could be attributed to oxidative stress or reactive oxygen species generation, impairing cell membrane integrity and microbial activity (Mullai et al. 2013).

### Sugar consumption and VFA production

**Fig. 7:**
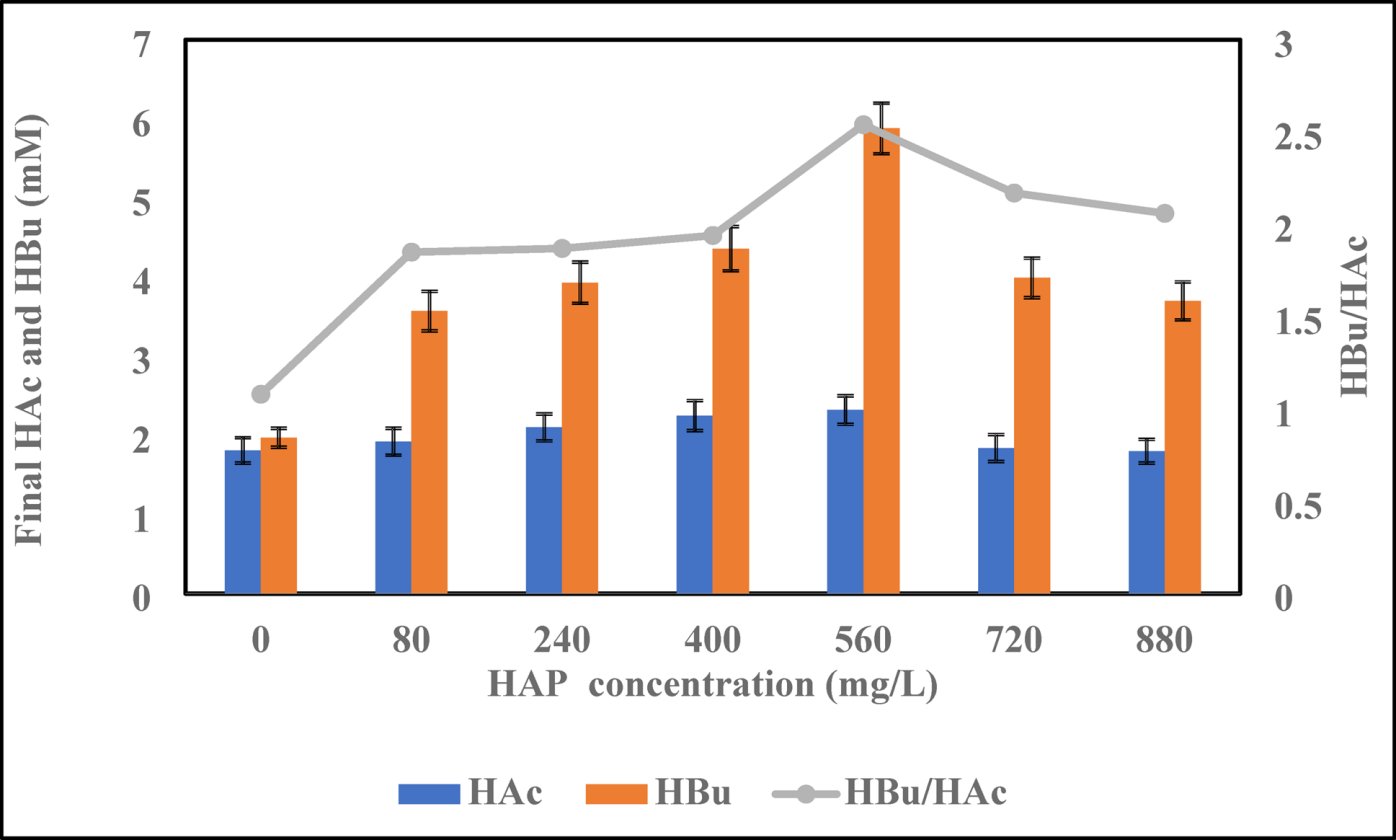
Effect of HAP on soluble metabolite production: final HAc and HBu concentrations (mean ± SD, n = 3) and the HBu/HAc ratio

In thermophilic dark fermentation, glucose was efficiently metabolized, resulting in the generation of soluble microbial products (SMPs). The addition of hydroxyapatite (HAP) significantly enhanced metabolite release from anaerobically digested biomass. As shown in Fig. 7, HAP supplementation led to a notable increase in volatile fatty acid (VFA) concentrations, primarily butyric acid (HBu) and acetic acid (HAc), indicating enhanced fermentation activity. At an optimal HAP concentration of 560 mg/L, the system achieved the highest concentrations of HBu (~ 21.5 mM) and HAc (~ 8.5 mM). This condition also corresponded to the maximum hydrogen yield of 1.085 mol H_2_/mol sugar, as recorded in Table 4, confirming a strong correlation between VFA production and biohydrogen generation. The HBu/HAc ratio peaked at ~ 2.5, signifying a metabolic preference for the butyrate-type fermentation pathway, which is known to favour hydrogen production.

This metabolic shift suggests the prevalence of hydrogenogenic bacteria, notably *Thermoanaerobacterium thermosaccharolyticum*, which preferentially utilize the butyrate route. The reactions associated with HAc- and HBu-type fermentations are presented below:

8 $$ {\text{C}}_{6} {\text{H}}_{12} {\text{O}}_{6} + {\text{H}}_{2} {\text{O}} \to 2{\text{CH}}_{3} {\text{COOH}} + 2{\text{CO}}_{2} + 4{\text{H}}_{2} $$

9 $$ {\text{C}}_{{6}} {\text{H}}_{{{12}}} {\text{O}}_{{6}} \to {\text{CH}}_{{3}} {\text{CH}}_{{2}} {\text{CH}}_{{2}} {\text{COOH}} + {\text{2CO}}_{{2}} + {\text{2H}}_{{2}} $$

Across all tested conditions, the HBu/HAc ratio remained > 1.8, confirming the dominance of the butyrate pathway (Gadhe et al. 2015).This shift is advantageous for hydrogen production and implies that HAP selectively enhanced microbial activity and steered fermentation toward butyrate dominance, likely by improving the availability of phosphate ions and promoting biofilm-like microbial aggregation. The pH values remained within the favorable range for anaerobic hydrogen production (4.01–4.71) across all experimental runs, suggesting the system’s tolerance to HAP addition (Table 4). Notably, no drastic acidification occurred, indicating microbial resilience and metabolic adaptation. The enhanced hydrogen production is attributed to two synergistic factors. Firstly, thermophilic conditions optimize hydrogenase enzyme activity, accelerating carbohydrate metabolism (Sun et al. 2021b). The second is the supplementation of hydroxyapatite, which promotes microbial enrichment, enhances substrate-to-product conversion, and likely improves electron transfer processes through calcium-phosphate interactions (Engliman et al. 2017).

Together, these findings confirm that HAP plays a dual role as a micronutrient carrier and metabolic modulator, improving hydrogen productivity by favouring a high-yield, butyrate-dominant fermentation route under thermophilic conditions.

### Microbial community analysis

The microbial community of the thermophilic consortium was analyzed to identify the dominant species that produce hydrogen. PCR amplification and 16S rRNA sequencing, followed by BLAST alignment, confirmed that all retrieved sequences belonged to the genus *Thermoanaerobacterium*. This indicates a 100% prevalence of this genus within the bacterial population. The community exclusively consisted of Firmicutes from family III, which aligns with earlier findings that this lineage often dominates thermophilic dark-fermentative systems (Jariyaboon et al. 2023). Table 6 revealed that the major species of *Thermoanaerobacterium* were similar to *Thermoanaerobacterium thermosaccharolyticum* (100%), *Thermoanaerobacterium bryantii* (98.5%), *Thermoanaerobacterium xylanolyticum* (98.25%), *Thermoanaerobacterium calidifontis* (98.25%), *Thermohydrogenium kirishiense* (98.25%), and *Thermoanaerobacterium aciditolerans* (98.25%). Recently, a thermophilic bacterium capable of creating hydrogen has been employed to improve biohydrogen production. *Thermoanaerobacterium* spp. are known to possess a wide range of polysaccharide-degrading enzymes, including amylase, cellulase, and xylanase, which enable efficient conversion of biomass to hydrogen via volatile fatty acid (VFA) intermediates (Haque et al. 2022; Huang et al. 2022; Okonkwo et al. 2020; Yin et al. 2020; Ersoy et al. 2023). According to a study (Pason et al. 2020), *T. thermosaccharolyticum* achieved 35.08 mol H_2_/g-substrate hydrogen yield through acetic and butyric acid pathways. Aside from that, study conducted by Zhang et al. (2011) was able to obtain 2.07 mol H_2_/mol glucose by utilizing a dominant W16 culture.

**Table 6:** Sequences Producing significant alignments from isolated sample culture by BLAST

No	Description	Total Score	Identical (%)	Accession
1	Thermoanaerobacterium thermosaccharolyticum strain CT6 16S ribosomal RNA gene, partial sequence	739	100	JX984971.1
2	Thermoanaerobacterium thermosaccharolyticum strain DJA2 16S ribosomal RNA gene, partial sequence	734	99.75	KJ831072.1
3	Thermoanaerobacterium thermosaccharolyticum strain CT72 16S ribosomal RNA gene, partial sequence	734	99.75	JX984968.1
4	Thermoanaerobacterium thermosaccharolyticum strain D120-70 16S ribosomal RNA gene, complete sequence	734	99.75	AF247003.1
5	Thermoanaerobacterium bryantii 16S ribosomal RNA gene, partial sequence	706	98.5	AY140670.1
6	Thermoanaerobacterium sp. strain PSU2 16S ribosomal RNA gene, partial sequence	701	98.25	KY407568.1
7	Thermoanaerobacterium xylanolyticum strain LX-11 16S ribosomal RNA, partial sequence	701	98.25	NR_102771.1
8	Thermoanaerobacterium calidifontis strain Rx1 16S ribosomal RNA, partial sequence	701	98.25	NR_113051.1
9	Thermoanaerobacterium xylanolyticum LX-11, complete genome	3505	98.25	CP002739.1
10	Thermohydrogenium kirishiense strain DSM 11055 16S ribosomal RNA, partial sequence	701	98.25	NR_117161.1
11	Thermoanaerobacterium aciditolerans strain 761–119 16S ribosomal RNA, partial sequence	701	98.25	NR_042856.1
12	Thermoanaerobacterium aotearoense strain SCUT27 chromosome, complete genome	3444	98	CP047602.1
13	Thermoanaerobacterium thermosaccharolyticum gene for 16S ribosomal RNA, partial sequence, strain: JCA-5603	695	98	LC127098.1

Previous studies have employed highly specific thermophilic strains such as *Thermoanaerobacterium thermosaccharolyticum* for biohydrogen production due to their ability to ferment a broad range of mono-, di-, and tri-saccharides, including both hexoses and pentoses (Litti et al. 2022). In one study, hydrogen production reached 2.47 mmol H_2_/L/h using this species (Arisht et al. 2022). In another investigation, the same bacterium achieved a yield of 0.208 mmol/L/h under different conditions.These findings demonstrate that *Thermoanaerobacterium* spp. possess a high hydrogenogenic potential and efficient substrate degradation capabilities, likely due to their robust enzymatic activity (Saripan et al. 2021). Therefore, the elevated hydrogen yield observed in this study may be attributed to the dominant presence of *Thermoanaerobacterium* spp. in the mixed culture.

## Conclusion

This study successfully synthesized stoichiometric hydroxyapatite (Ca/P = 1.67) from eggshell waste under optimized conditions (pH 10.5, 950 °C, 2 h). When applied as a bio-additive in thermophilic fermentation, HAP significantly enhanced hydrogen production, achieving a 59.73% increase in yield at 560 mg/L. The enhancement was linked to improved microbial activity, biomass accumulation, and a shift toward butyrate-type fermentation dominated by *Thermoanaerobacterium thermosaccharolyticum*. These findings confirm HAP’s dual role as a micronutrient source and metabolic enhancer, supporting its potential for sustainable biohydrogen production.

## Supplementary Information

**Additional file 1**.

## Data Availability

Data will be made available on request.
