# Supplementary material for: Thermophilic fermentation enhanced by eggshell-derived hydroxyapatite for sustainable hydrogen production
Source: Bioresour Bioprocess. 2025 Nov 21;12(1):136. doi: 10.1186/s40643-025-00976-4 (PMC12638588; doi:10.1186/s40643-025-00976-4)
Supplement: Supplementary file 1 — Additional file 1. [file 40643_2025_976_MOESM1_ESM.docx]

**Supplementary Material**


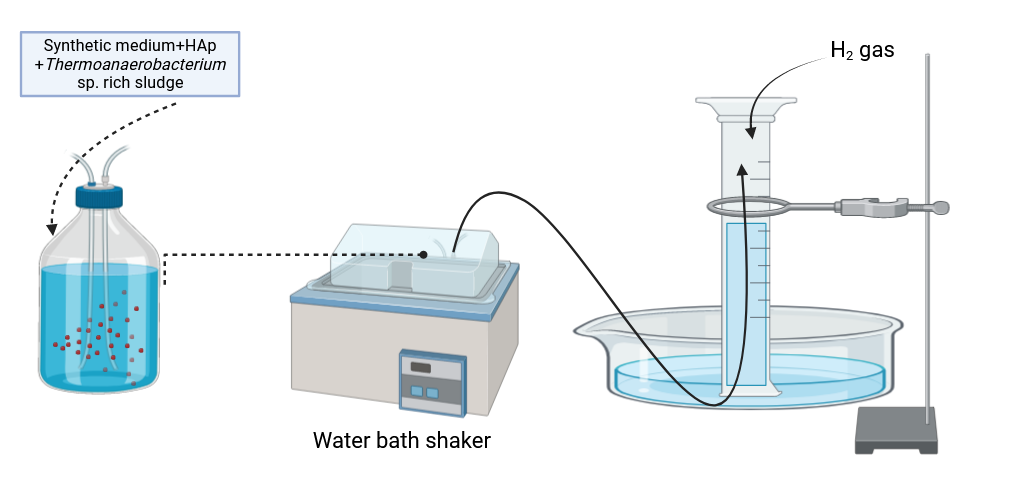


Figure S1. Schematic representation of the thermophilic dark fermentation experimental setup.
